# Relationship between Lipoprotein (a) and cognitive function – Results from the Berlin Aging Study II

**DOI:** 10.1038/s41598-020-66783-3

**Published:** 2020-06-30

**Authors:** Franziska Röhr, Nina Bucholtz, Sarah Toepfer, Kristina Norman, Dominik Spira, Elisabeth Steinhagen-Thiessen, Christina M. Lill, Lars Bertram, Ilja Demuth, Nikolaus Buchmann, Sandra Düzel

**Affiliations:** 1 Charité – Universitätsmedizin Berlin, corporate member of Freie Universität Berlin, Humboldt-Universität zu Berlin, and Berlin Institute of Health, Department of Endocrinology and Metabolism, 10117 Berlin, Germany; 2 German Institute of Human Nutrition, Department of Nutrition and Gerontology, Potsdam-Rehbruecke (DIfE), Nuthetal, Germany; 3 Charite - Universitätsmedizin Berlin, Forschungsgruppe Geriatrie am EGZB, Berlin, Berlin, Germany; 4 Section for Translational Surgical Oncology and Biobanking, Department of Surgery, University of Lübeck and University Medical Center Schleswig-Holstein, Campus Lübeck, 23552 Lübeck, Germany; 5 Ageing Epidemiology Research Unit, School of Public Health, Imperial College, London, SW71 UK; 6 Lübeck Interdisciplinary Platform for Genome Analytics, Institutes of Neurogenetics and Cardiogenetics, University of Lübeck, Lübeck, Germany; 7 Center for Lifespan Changes in Brain and Cognition (LCBC), Department of Psychology, University of Oslo, Oslo, Norway; 8 Charité - Universitätsmedizin Berlin, BCRT - Berlin Institute of Health Center for Regenerative Therapies, Berlin, Germany; 9 Department of Cardiology, Charité - University Medicine Berlin (Campus Benjamin Franklin), Berlin, Germany; 10 Max Planck Institute for Human Development, Berlin, Germany

**Keywords:** Psychology, Diseases, Health care, Medical research, Pathogenesis, Risk factors

## Abstract

It has been suggested that an age-related loss of cognitive function might be driven by atherosclerotic effects associated with altered lipid patterns. However, the relationship between Lipoprotein (a) [Lp(a)] and healthy cognitive aging has not yet been sufficiently investigated. For the current analysis we used the cross-sectional data of 1,380 Berlin Aging Study II (BASE-II) participants aged 60 years and older (52.2% women, mean age 68 ± 4 years). We employed the Consortium to Establish a Registry for Alzheimer’s Disease (CERAD)-Plus test battery to establish latent factors representing continuous measures of domain specific cognitive functions. Regression models adjusted for *APOE* genotypes, lipid parameters and other risk factors for cognitive impairment were applied to assess the association between Lp(a) and performance in specific cognitive domains. Men within the lowest Lp(a)-quintile showed better cognitive performance in the cognitive domain executive functions and processing speed (p = 0.027). No significant results were observed in women. The results of the current analysis of predominantly healthy BASE-II participants point towards an association between low Lp(a) concentrations and better cognitive performance. However, evidence for this relationship resulting from the current analysis and the employment of a differentiated cognitive assessment is rather weak.

## Introduction

The prevalence and incidence of dementia and cognitive impairment are increasing with advancing age^1,2^. Decline of cognitive functioning is a health risk, personally resulting in significant socio-economic and medical consequences^1,2^. The trajectories of cognitive decline in healthy aging can vary, affecting cognitive domains differently. The best studied and presumably most relevant modifiable risk factors for cognitive decline are e.g. hypertension, diabetes mellitus, obesity, and smoking habits^3^. To date, early detection and treatment of these specific risk profiles seem to be the most promising approach so far to prevent or delay age-related cognitive decline or dementia^2,4^. Moreover, an association between plasma lipid levels (low-density-lipoprotein (LDL) cholesterol, high-density-lipoprotein (HDL) cholesterol, apolipoprotein E4 (APOE4) and Lipoprotein(a) [Lp(a)]) with cognitive function has been reported. In particular, atherosclerosis and cerebrovascular diseases which are favored by altered lipid concentrations are discussed to promote cognitive impairment, including e.g. Alzheimer´s disease or vascular dementia [reviewed in^5^]^3^. So far, findings regarding the association between blood lipid levels and cognition are contradictory. Besides that, there is no yet common or internationally standardized assessment used within these analyses to define and evaluate different domains of cognitive function.

With regard to the relationship between metabolic markers and cognitive functioning, Van den Kommer and colleagues analyzed data from the Longitudinal Aging Study Amsterdam and reported a link between low LDL-C and worse cognitive performance in general. They also found a faster decline in processing speed in subjects with low LDL-C. Additionally, high HDL-C concentrations were found to be associated with better memory performance^6^. Ancelin *et al*. defined specific cognitive domains (visual memory, verbal fluency, psychomotor speed and executive abilities) and found associations between high total cholesterol, low HDL-C, high LDL-C and the risk of cognitive decline in psychomotor speed, executive abilities, and verbal fluency, but in men only^7^. In contrast, lower performance in motor speed was found to be associated with high HDL-C. Lower executive abilities were linked to low LDL-C and triglycerides in women^7^.

With respect to the relationship between Lp(a) and cognitive function, current studies reported conflicting results.

Lp(a) consists of an LDL-like particle, which is covalently attached to the apo(a) protein and differs structurally from other apolipoproteins. Its serum concentration is mainly genetically determined and its physiological function is still relatively unknown^8,9^.

Results from observational studies and studies employing mendelian randomization suggest a causal association between Lp(a) and cardiovascular diseases, e.g. myocardial infarction, stroke, and aortic valve stenosis and metabolic health^10–12^.

Notably, higher Lp(a) concentrations have also been linked to positive outcomes such as lower incidence of diabetes or better pulmonary function^13–17^.

A majority of studies found a link between higher Lp(a) concentrations and vascular dementia (VD)^18,19^ or Alzheimer’s disease (AD)^20^, thus interpreting an increased occurrence of cardiovascular diseases, ischemia and inflammation promoted by Lp(a) as a possible mechanism for cerebrovascular disease and cognitive decline. Solfrizzi *et al*.^20^ discovered a non-linear relationship between Lp(a) and AD, whilst study participants over 72 years and high Lp(a) levels showing a reduced risk to develop AD. Iwamoto *et al*. suggested an inverse effect were high Lp(a) levels being associated to an increased risk of VD but an decreased occurrence of AD^18^. In contrast, Kunutsor *et al*. even reported evidence for a protective effect of Lp(a) on cognitive decline in general^21^. Other authors found no association of elevated Lp(a) levels with poorer cognitive performances at all^22^.

It also has been shown that the combination of high Lp(a) plasma level and carrier status of the apolipoprotein E (*APOE*) epsilon 4 allele increases the risk for late-onset Alzheimer´s disease, while Lp(a) might protect against this decline in *APOE*-4 non-carriers^23^.

Taken together, the mechanisms for conflicting cross-sectional results on the relationship between Lp(a) and cognitive performance are still unclear. Existing studies mainly focus on advanced stages of cognitive decline, e.g. dementia or AD, and mostly not differentiate between cognitive domains. Moreover, studies investigating sex-specific links and differences between Lp(a) and cognitive performance are sparse.

To address these limitations and shed more light in investigating the associations of Lp(a) on different cognitive abilities, we employed the Consortium to Establish a Registry for Alzheimer’s Disease (CERAD)-Plus test battery^24^. The German CERAD-Plus test battery contains various tasks for assessing different cognitive abilities. Existing studies that investigated the factor structure of the CERAD test battery using exploratory factor analyses (EFA) show inconsistent results. While Strauss and Fritsch found a general factor, Morris *et al*. (1989) reported a three factor structure, whereas Collie *et al*. (1999) found even five factors of selected CERAD subtest^25^. It is important to mention that all studies differ in the samples selected (Alzheimer’s patients and healthy adults) and in the selection of CERAD subtest which makes it hard to compare the resulting factor structures. While Morris *et al*. (1989), for example, included the total score of MMST and the word list learning, Collie *et al*. (1999) only chose individual subtests of single tests of the MMST^25^. Strauss and Fritsch (2004) also used subtests of the MMST, whereby the composition of the subtests in Collie *et al*. (1999) differs^25,26^. In addition, the CERAD items have so far only been examined using EFA but the theoretical assumptions about different cognitive domains or specific relationships between factors have not taken into account. In addition, the German version of CERAD Plus includes novel sub-tests, namely trail making test A & B and phonemic word fluency test. These tasks have not yet been included in the factor analyses mentioned above. Taken together, the factor structure of the complete CERAD items in a large sample of healthy older adults remains unclear.

To this end, we carried out exploratory and confirmatory factor analyses which resulted in establishing four latent cognitive factors representing different domain specific cognitive functions in a large sample of 1,380 older and generally healthy Berlin Aging Study II (BASE-II) participants. We hypothesized that elevated Lp(a) levels are associated with poorer cognitive performance in specific domains that might be vulnerable to unfavorable metabolic and cardiovascular risk profile in older males and females, such as executive functions working memory and episodic memory.

## Methods

### Participants

The Berlin Aging Study II (BASE-II) aims to identify factors involved in ‘healthy’ and ‘unhealthy’ aging and collected medical baseline data of 2,171 participants (≈75% aged 60–84 years and ≈25% aged 20–37 years) between 2009 and 2014. The participants comprise a convenient sample of community-dwelling participants, living in the greater metropolitan area of Berlin, Germany, and the collected data cover numerous ageing-relevant variables^27,28^. All participants scored more than 27 points on the MMSE^29^. All participants gave written informed consent to participation and the Ethics Committee of the Charité-Universitätsmedizin Berlin approved this study (approval number EA2/029/09) and all research was performed in accordance with relevant guidelines/regulations.

### Laboratory tests

Blood samples were drawn after a fasting period of at least 8 hours. Plasma concentration of Lp(a) was assessed using an enzyme-linked immunosorbent assay. Triglycerides were quantitatively acquired photometrically by an enzymatic *in vitro* test using the analyses instrument of Roche. Total cholesterol (TC), low density lipoprotein cholesterol (LDL-C), and high-density lipoprotein cholesterol (HDL-C) were determined in the same way. HbA1c was measured using high-performance chromatography (Variant II Turbo HbA1c Kit- 2.0, Bio-Rad). The thyroid stimulating hormone (TSH), Vitamin B12, folic acid and the concentration of C-reactive protein (CRP) were measured by an electrochemiluminescence immunoassay. Magnesium was measured photometrically by using a xylidyl blue complex. Sodium and potassium were determined by an ion-selective electrode. Homocystein was measured photometrically by an enzyme-cycling assay. *APOE* genotyping was performed for two polymorphisms defining the epsilon 2/3/4 haplotype from blood-derived DNA samples either by direct sequencing (performed at LGC Genomics, Berlin, Germany) or by targeted genotyping using TaqMan assays (ThermoFisher Scientific, Foster City, CA) on a QuantStudio-12K-Flex system in 384-well format. *APOE* haplotypes were then classified according to the “epsilon 2/3/4” allele designation derived from polymorphisms rs7412 [a.k.a. as “epsilon 2-allele”] and rs429358 [a.k.a. “epsilon 4-allele]).

### Co-variables

Regular alcohol intake (yes/no) and current smoking status (yes/no) were evaluated by standardized questions. Information on past and current diseases was obtained from participant reports, clinical examinations and laboratory tests. Diagnoses were used to compute a morbidity index largely based on the categories of the Charlson index, which is a weighted sum of moderate to severe, mostly chronic physical illnesses, including cardiovascular (e.g., congestive heart failure), cancer (e.g., lymphoma), and metabolic diseases (e.g., diabetes mellitus)^30,31^. We used the Rapid Assessment of Physical Activity (RAPA) questionnaire to assess physical activity of the study participants^32^.

As a screening for depression we employed the geriatric depression scale (GDS)^33^.

### Neuropsychological assessment

To assess cognitive performance the German version of the neuropsychological test battery CERAD-Plus (Consortium to Establish a Registry for Alzheimer’s Disease) was applied^24^. The complete test battery was administered in individual sessions to all BASE-II participants of the older group studied here. The following nine CERAD-Plus (age, gender, education adjusted z-values) scores were finally used to evaluate the cognitive performance of the subjects: Word list learning, word list recall, word list recognition, recall the figure, copying a figure, responses of semantic fluency, phonemic fluency, Trail Making Test A and Trail Making Test B.

### Behavioral data analysis

#### Exploratory factor analyses (EFA) of CERAD-Plus test

In a first set of analyses we aimed to explore the factor structure of CERAD-Plus in our large BASE-II sample consisting of healthy older adults by applying EFA. In this first set of analyses we carried out a principal component analyses (PCA) in SPSS based on the age, education and gender-corrected z-values of the following eleven CERAD-Plus tests: word list learning, word list recall, word list recognition, recall the figure, copying a figure, semantic fluency, phonemic fluency, Trail Making Test A and Trail Making Test B, Boston naming test, and word list intrusions.

#### Confirmatory factor analyses (CFA) of CERAD test

In order to investigate whether CERAD tests form the hypothezised specific cognitive domains we selected the remaining nine CERAD subtest and covariates according to our hypothesis and applied CFA before using the extracted factor scores for conducting regression analyses. CFA allows testing structural hypotheses about associations among multiple variables by examining how well a given model is able to reproduce the variance–covariance matrix of a set of observed variables. Factor analysis represents the variance shared by the observed (measured) variables. The latent variables can be assumed to be free of task-specific sources of variance as well as measurement error. Previous studies demonstrated specific factor structures of the CERAD tests^24,26^.

In the current study, latent factor models were established by using MPlus v6.1^34^ We relied on standard indices such as the Root Mean Square Error of Approximation (RMSEA) and the Comparative Fit Index (CFI) for evaluation of model fit. Commonly accepted thresholds indicating good model fit are 0 < =RMSEA < = 0.05 and 0.97 < =CFI < = 1^35^.

We performed CFA to tests weather previous suggested factor models fit to our data: (A) a general latent factor model on which all nine CERAD-Plus tasks simultaneously loaded on^24,26^, (B) a two latent factor model which is based on the theoretical differentiation of MCI in amestic (word list learning, word list recall, word list recognition, recall the figure) and non-amnestic (semantic fluency, phonemic fluency, Trail Making Test A and Trail Making Test B) state, (C) a three latent factor model (verbal memory, visuo-construction, executive functions and processing speed) based on the results of Morris *et al*.^24^ and (D) in a four latent factor (verbal memory, visuo-construction, executive functions and processing speed, verbal fluency) model that was suggested by our exploratory factor analyses (PCA, Supplementary Table 7). For details regarding model comparisons and fit indices, see Table 1.

**Table 1:** Communalities (h²) of the nine CERAD subtests of CFA models 1–4.

domain/test	factor loadings (standardized)			
	general factor model	2 factor model	3 factor model	4 factor model
verbal memory/word list learning	0.771**	0.749**	0.732**	0.730**
verbal memory/word list recall	0.823**	0.889**	0.915**	0.905**
verbal memory/word list recognition	0.422**	0.432**	0.428**	0.424**
visuo-construction/copying a figure	0.091**	−0.004	0.466**	0.491**
visuo-construction/recall the figure	0.306**	0.272**	0.980**	0.932**
verbal fluency/semantic fluency	0.371**	0.497**	0.477**	0.613**
verbal fluency/phonemic fluency	0.349**	0.486**	0.460**	0.491**
executive functions & processing speed/Trail Making Test part A	−0.210**	−0.449**	−0.517**	0.526**
executive functions & processing speed/Trail Making Test part B	−0.340**	−0.648**	−0.647**	0.847**

*CERAD subtests:* encode = word list learning; recall = word list recall; recogn = word list recognition; frecall = recall the figure; fcopy = copying a figure; semantic = responses of the semantic fluency; phonem = phonemic fluency; TMTA = Trail Making Test part A; TMTB Trail Making Test part B, **p < 0.001.

Each latent factor was defined by at least two indicators, namely the sum of correct responses from word list learning, word list recall, word list recognition (3 indicators) for the verbal memory factor, the sum of correct responses of copying a figure and recall the figure (2 indicators) for the visuo-construction factor, the number of correct responses of the semantic fluency and the phonemic fluency task (2 indicators) for defining the verbal fluency factor, and the score of time in seconds spend to finish the Trail Making Test part A and B (2 indicators) for the executive functions and processing speed factor.

#### Factor scores of the four factor model (D)

**Figure 1:**
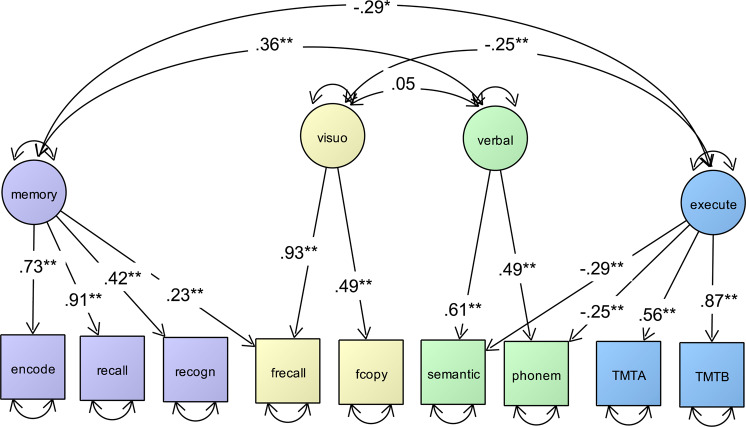
Depiction of a simplified CFA of the final selected four latent factor model of CERAD with good model fit (χ² 71.102, DF = 20; RMSEA = 0.031; CFI = 0.979) including standardized factor loadings (single headed arrows) and covariances between latent factors (double headed arrows). Circles represent latent factors and squares represent manifest variables. Double-headed arrows with both heads pointing on a manifest variable represent the variance of this variable. Latent factors: memory = verbal memory; visuo = visuo-construction; verbal = verbal fluency; execute = executive functions and processing speed. Manifest variables: encode = word list learning; recall = word list recall; recogn = word list recognition; frecall = recall the figure; fcopy = copying a figure; semantic = responses of the semantic fluency; phonem = phonemic fluency; TMTA = Trail Making Test part A; TMTB Trail Making Test part B, **p < 0.001.

For each participant the four individual intercorrelated factor scores of the best fitting model (D) were estimated using the regression method (modal posterior estimator) in Mplus v6.1 (Muthén & Muthén, 2007) on the basis of the final selected model D (Fig. 1)^34^.

We applied the extracted individual four factor scores as independent variables for subsequent regression analyses within our sample.

The data was analyzed using the Statistical Package for Social Science (SPSS, IBM Analytics) version 25. Mann-U-test was performed to assess differences between continuous data. Comparisons between groups were performed applying Chi^2^-test. Adjusted regression models were performed to estimate whether Lp(a) is associated with four cognitive factor scores. According to the EAS consensus panel Lp(a) levels in the upper quintile can be considered as elevated with respect to its role as a cardiovascular risk factor^11^ and it has become current practice in the field to examine Lp(a) by quintiles. Following this, we divided Lp(a) in quintiles and low Lp(a)-concentrations (Lp(a) quintile 1) were used as an independent variable. During the data quality check less than 15 unrealistically high or low values, especially for potassium, were excluded from the analyses (for example attributed to preanalytical problems and due to low plausibility).

All reported associations were stepwise controlled for age, weight, height, HbA1c and APOE epsilon 4 carrier status (i.e. participants carrying the APOE 24, APOE 34 or APOE 44) (model1), additionally for regular alcohol intake, current smoking, physical activity level (RAPA), depression (GDS-Score), TSH, homocysteine and CRP (model 2) as depression, endocrinopathies and inflammation are known to mimic or rather promote cognitive deficits. Since vitamin deficiencies, and shifted electrolytes are also known to affect cognitive performance we additionally considered vitamin B12, folic acid, sodium, potassium and magnesium as covariates in model 3, also adjusting for the morbidity burden (morbidity index). Due to the genetic determination of Lp(a), which can hardly be influenced by other factors – and which has no influence on the co-variates used here -, we considered all the co-variables used as confounders and assumed no mediation effect here.

P-values of <0.05 were considered to indicate statistical significance. Additionally, we applied Bonferroni correction to account for multiple testing (four latent factors of cognitive performance), therefore p-values below 0.05/4 = 0.0125 were considered to be statistically significant.

## Results

Cross-sectional data for Lp(a), *APOE* epsilon 4 carrier status and the four latent factor scores estimated here and reflecting cognitive functions of different domains (verbal memory, visuo-construction, executive functions and processing speed, verbal fluency) were available for 1,380 old BASE-II participants (mean age 68 ± 4 years; 52.2% women).

Baseline characteristics of the study population are displayed in Table 2, divided by sex. Due to known sex-specific differences in Lp(a) levels,^36–38^, in brain development^39^ and metabolism^40,41^, we performed sex-specific analyses. Men were significantly more frequently current smokers, reported regular alcohol intake more often, and were less frequently physically active. Regarding lipid patterns, men had significantly lower concentrations of HDL-C, LDL-C and Lp(a), but increased levels of serum triglycerides, when compared to women.

**Table 2:** Characteristics of the BASE-II study population divided by sex.

	Men (n = 659)	Women (n = 721)	p-value
Age [years]	68 ± 3.6	68 ± 3.4	<0.001
Weight [kg]	83.95 ± 12.27	69.77 ± 12.34	<0.001
Height [cm]	175.55 ± 6.21	162.83 ± 6.02	<0.001
BMI [kg/m^2^]	27.23 ± 3.65	26.34 ± 4.62	<0.001
Regular alcohol intake ^a^	598 (91.0)	633 (87.8)	0.055
Current smoking ^a^	73(11.1)	63 (8.8)	<0.001
Physically inactive ^a^	73 (11.1)	56 (7.8)	0.04
GDS score	24.61 (1.04)	24.63 (1.05)	n.s.
**Cognitive function (factor scores)**.			
Verbal memory	−0.1805 ± 0.9406	0.2259 ± 0.8751	<0.001
Visuo-construction	0.1460 ± 0.9589	−0.1276 ± 0.9381	<0.001
Executive functions and processing speed	0.0505 ± 0.9427	−0.0708 ± 0.8245	0.032
Verbal fluency	−0.1015 ± 0.7355	0.1018 ± 0.7065	<0.001
**Lipid profile**			
Total Cholesterol [mg/dL]	202 ± 38	226 ± 38	<0.001
HDL-C [mg/dL]	55 ± 14	69 ± 16	<0.001
LDL-C [mg/dL]	123 ± 34	136 ± 35	<0.001
TG [mg/dL]	122 ± 77	104 ± 51	<0.001
Lp(a) [mg/L]	230.2 ± 320.6	265 ± 338.5	0.004
***APOE*** **genotypes**^**a**^			
22	5 (0.8)	3 (0.4)	n.s.
23	89 (13.5)	102 (14.1)	n.s.
24	15 (2.3)	25 (3.5)	n.s.
33	394 (59.8)	441 (61.2)	n.s.
34	146 (22.2)	138 (19.1)	n.s.
44	10 (1.5)	12 (1.7)	n.s.
Vitamin B12 [ng/L]	371.8 ± 213.5	423.1 ± 293.8	<0.001
Homocysteine [µmol/L]	14.29 ± 3.99	12.49 ± 3.44	<0.001
CRP [mg/L]	1.8 ± 2.4	2.2 ± 3.4	0.015
Folic acid [µg/L]	10.88 ± 5.34	11.97 ± 5.77	<0.001
HbA1c [%]	5.7 ± 0.6	5.6 ± 0.5	0.012
TSH basal [mU/L]	2.10 ± 3.07	2.29 ± 4.2	0.034
Sodium [mmol/L]	139 ± 3	140 ± 3	0.005
Potassium [mmol/L]	4.5 ± 0.4	4.5 ± 0.4	n.s.
Magnesium [mmol/L]	0.82 ± 0.07	0.81 ± 0.07	n.s.

BMI = body mass index; GDS = geriatric depression scale; HDL-C = high density lipoprotein cholesterol; LDL-C = low density lipoprotein cholesterol; Lp(a) = lipoprotein(a), *APOE* = apolipoprotein E; CRP = C-reactive protein; HbA1c =hemoglobin A 1c; TSH = thyroid stimulating hormone.

Mann-U-test was performed to assess differences between continuous data. Comparisons between groups were performed applying Chi-squared- test (^a^).

The exploratory PCA including all CERAD subtests resulted in one to five factor model solutions. Only the three to five factor models showed a good model fit (see supplementary table 6). By inspecting the factor loadings, the indicators Boston naming test, and word list intrusion did not load significantly (all p’s > 0.05) on the factors. As a consequence we excluded the two tests from the PCA, resulting in one-four factor model solutions which are summarized in supplementary table 7. The best model fit showed model 4 consisting of a verbal memory factor, a visuo-construction factor, an executive functions and processing speed factor and a verbal fluency factor.

Next, by applying CFA we established four different latent factor models based on previous PCA findings and theoretical assumptions reported in the literature. Model D provided the best fit to the data [χ20 = 71.102, CFI = 0.979, RMSEA = 0.031], relative to the global (model A), two (model B) and three-factor models (Model C; see Table 4 for fit indices of model comparison).

We used model D subsequent analyses. Core results of the CFA for model D are shown in Fig. 1 were we specified four intercorrelated latent variables, each with unique loadings from the corresponding nine CERAD-Plus test scores (Fig. 1). All items loaded reliably on the postulated latent factors (p < 0.001).

Next, we extracted the four factor scores from model D and used them as independent variables for subsequent analyses. Factor scores are z-standardized composite variables which provide information about an individual’s placement on the factors. For factor 1 (verbal memory; mean = 0.00; SD = 0.91; range = −3.69–2.46) the higher the standardized score the better is the performance within this domain. The same direction applies for factor 2 (visuo-construction; mean = 0.00, SD = 0.86, range = −2.45–2.15), and factor 4 (verbal fluency; mean = 0.00, SD = 0.73, range = −2.51–2.45). In contrast, a high factor score on factor 3 (executive functions and processing speed; mean = 0.00, SD = 0.93, range = −2.53–3.82), reflects a lower performance.

By inspecting the extracted individual four cognitive factors scores, women performed significantly better in tests related to verbal memory, executive functions and processing speed and verbal fluency, while men showed better results in tests representing visuo-construction (see Table 2). As shown in supplementary tables 1 and 2, we found no significant difference between cognitive functions and Lp(a) levels in men and women in unadjusted calculations.

With respect to other covariates, which may be linked to cognitive performance, men had lower concentrations of vitamin B12 and folic acid. Moreover, men showed higher levels of homocysteine, higher concentrations of HbA1c and higher BMI. Electrolyte levels and TSH concentrations were equally distributed, independent of sex. Notably, CRP concentrations were higher in women.

Next, we set up different linear regression models assessing the association between Lp(a) and the four latent factors for cognitive functions, namely verbal memory, visuo-construction, executive functions and processing speed, and verbal fluency. Lp(a) was divided in quintiles and low Lp(a)-concentrations (Lp(a) quintile 1) were used as an independent variable. Table 3 shows the results of three linear regression models adjusted for an increasing number of confounders with the factor scores for executive functions and processing speed as the dependent variable.

**Table 3:** Association between Lp(a) and the latent factor score reflecting *executive functions and processing speed* divided by sex including lipid-lowering agents.

	Men				Women			
	beta	SE	p-value	R^2*^	beta	SE	p-value	R^2*^
Model 1	−0.178	0.088	0.043	0.049	−0.093	0.080	0.246	0.045
Model 2	−0.202	0.092	0.029	0.056	−0.087	0.084	0.301	0.040
Model 3	−0.217	0.098	0.027	0.050	−0.110	0.089	0.215	0.039
Model 4	−0.223	0.098	0.023	0.048	−0.110	0.088	0.211	0.045
Model 5	−0.221	0.098	0.025	0.047	−0.124	0.087	0.155	0.057
Model 6	−0.215	0.098	0.028	0.048	−0.103	0.088	0.241	0.049

Model 1: age, weight, height, HbA1c, APOE genotype.

Model 2: Model 1 + regular alcohol intake, current smoking, physical inactivity, GDS score, TSH, homocysteine, CRP.

Model 3: Model 2 +vitamin B12, magnesium, potassium, sodium, folic acid, morbidity index.

Model 4: Model 3 + HDL-C + lipid-lowering agents.

Model 5: Model 3 + LDL –C + lipid-lowering agents.

Model 6: Model 3 + TG + lipid-lowering agents.

GDS = geriatric depression scale; HDL-C = high density lipoprotein cholesterol; LDL-C = low density lipoprotein cholesterol; TG = triglycerides, Lp(a) = lipoprotein(a), APOE = apolipoprotein E; CRP = C-reactive protein; HbA1c = hemoglobin A 1c; TSH = thyroid stimulating hormone,*= R2 corrected, p-values <0.05 = statistical significant, corrected p-value (Bonferroni).

<0.0125 = statistical significant.

**Table 4:** CFA results of the model comparisons and fit indices testing different latent factor solutions of the 11 CERAD-Plus indices.

Model	χ²	df	p	RMSEA	CFI
A (1 factor)	954.792	27	<0.001	0.149	0.613
B (2 factors)	708.014	25	<0.001	0.132	0.715
C (3 factors)	271.416	23	<0.001	0.083	0.897
D (4 factors)	71.102	20	<0.001	0.031	0.979

CFA = Confirmatory factor analyses; CFI = comparative fit index; RMSEA = root mean square error of approximation.

In the fully adjusted model 3 we found a nominal statistically significant association between low Lp(a) levels and the factor score of executive functions and processing speed (beta: −0.217; SE: 0.098; p = 0.027, R^2^ = 0.050) in men; in women, however, this association was not detected. Following correction for multiple testing (testing the Lp(a) association with each of the four latent factors of cognitive performance), this association found in men was no longer statistically significant. A recalculation of this model including sedatives as an additional co-variable did not change these results significantly (0.220 SE: 0.096; p = 0.023, R^2^ = 0.052).

We recalculated model 3 adding HDL-C (model 4), LDL-C (model 5) and triglycerides (model 6) separately as independent variables, and also included the use of lipid lowering medication as a co-variable in the regression analyses. Adjustment for these other lipid parameters did not change the results significantly. The supplementary table 3 summarizes the results of the calculated and fully adjusted models investigating the relationship between Lp(a) and the four latent factor scores for cognitive function established in the current study. Except the above described association between Lp(a) and executive functions and processing speed in older men, no nominal association was found between Lp(a) and the three other cognitive domains studied here. As the *APOE* epsilon 4 allele is a risk factor for the development of AD, we additionally recalculated model 3 (see supplementary table 4) excluding subjects with *APOE* epsilon 4 alleles, this, however, did not change the results significantly.

## Discussion

In the current analysis of 1,380 older community-dwelling participants of BASE-II, low plasma concentrations of Lp(a) were associated with better cognitive performance in executive functions and processing speed in men only at nominal significance. This association was not found in women. This association was independent of other lipid parameters, *APOE* genotype and potential confounders. Moreover, there was no association between single markers of LDL-C, HDL-C and triglycerides with cognitive function.

This is to our knowledge the first study that established a four latent factor-structure including all CERAD-Plus based tests and investigated the associations between the four resulting cognitive domains to Lp(a) in a predominantly healthy large sample of older adults. Albeit after correcting for multiple testing none of the results remained significant, our analyses point towards a possible sex-specific link between Lp(a) and executive functions and processing speed.

Our finding is in line with other studies that have been shown that an optimal control of cardiovascular risk factors may reduce the risk of cognitive decline^42^, whilst high levels of Lp(a) increase the atherothrombotic risk by various mechanisms such as impaired fibrinolysis, increased cholesterol deposition in arterial walls and by stimulating inflammatory processes at the vascular walls^43,44^. From this pathogenic viewpoint, a link between Lp(a) and impaired cognitive performance remains plausible. In addition, stroke is an established risk factor for all-cause dementia^45^ and elevated Lp(a) is an independent risk factor for stroke^46^.

Further, Lp(a) levels seem to be significantly higher in both men and women with coronary artery disease compared to those without^47^ and small-vessel disease promoted by elevated Lp(a) may induce microstructural alterations leading to brain damage and poorer cognitive function^9–12^.

Thus, the current results may indicate a preclinical/prodromal stage of sex-specific vascular-related cognitive decline. A sex-specific difference seems plausible, as atherosclerotic manifestations affect men earlier in life, although increased Lp(a) concentrations are observed in postmenopausal women due to hormonal changes^36–38^.

Premenopausal women commonly have a less proatherogenic plasma lipid pattern than men, although physiological alterations regarding hormones (due to the menstrual cycle or menopause) do not affect lipid homeostasis significantly^48^. Moreover, there are sex differences in insulin sensitivity of glucose metabolism in the liver and muscle and insulin is an important regulator of lipid metabolism^48^. Women seem to be more sensitive to insulin regarding to glucose metabolism, whereas there are no differences between men and women in lipolysis^49^.

The generalizability of the results to the general population is limited by the convenient sampling of the BASE-II participants. This led to a bias of recruiting cognitively and physically high functioning individuals with relatively low incidence of comorbidity, especially cardiovascular diseases^27^ This may also drive the weak significant associations found in this study between cognition and Lp(a).

Additionally, the comparability of our results to previous studies is limited due to the differences in measures of cognitive functioning and decline. We aimed to investigate cognitive domains in a widely used measure such as CERAD. To our knowledge there is no internationally standardized assessment of cognitive function. Moreover, assessment of e.g. smoking habits or physical activity are mainly based on questionnaires, which might have led to over-or underreporting.

On the other hand the measurement of cognitive function employing the CERAD-Plus test battery followed by extracting latent factor scores representing different cognitive functions is a strength of the current study, however, this also raises the question about the comparability of the cognitive variables assessed in other studies, because the relations might depend on the selected cohort tests and cohorts.

Executive functions (EF) include a range of cognitive skills that facilitate purposeful, goal – directed and socially-competent behavior^50^. The three core EFs are “inhibition (inhibitory control, including self-control) and interference control” (selective attention and cognitive inhibition), “working memory” (holding information in mind and mentally working with; includes verbal working memory and visual-spatial working memory) and “cognitive flexibility” (changing perspectives spatially or interpersonally)^50^. Processing speed describes the efficiency an individual is able to perceive and act upon a stimulus^51,52^. Six variables have been described to assess the processing speed: “decision speed” (time to respond in cognitive tests with complex content), “perceptual speed” (time to respond in cognitive tests with simple content), “psychomotor speed” (simple tasks like drawing lines), “reaction time” (choice reaction time with visual stimuli and manual keypress responses), “psycho-physical speed” (decision accuracy with visual or auditory stimuli) and “time course of internal responses” (event-related potential)^52^.

The association (at nominal significance) between Lp(a), executive functions and processing speed found in the male group only of our dataset is in line with two theories with respect to deterioration of processing speed in older individuals the so-called “processing speed theory”^53^ and the “prefrontal-executive theory”^54^. On the one hand, diffuse age-related deterioration of white matter may result in the slowing of processing speed, on the other hand, structural age-related alterations of grey and white matter in the prefrontal cortex, which also has been associated with executive functions and processing speed might be responsible for worse performance in this cognitive domain. Both theories have been supported by neuroimaging studies^55^. An exact mechanism such as brain integrity through which Lp(a) may effect this specific cognitive domain has not been in the focus of the current study. However, an association between white matter lesions and atherosclerosis has been described elsewhere^56^. We think that white and gray matter integrity should be subject of further research, when investigating the association between Lp(a) and cognitive performance, although, it is currently unknown if Lp(a) can cross the blood brain barrier^57^, which may enable a direct influence of elevated Lp(a) blood levels on cognitive functioning.

Considering the inconsistent study results with respect to the association between Lp(a) and cognitive function (see supplementary table 5) the approach of investigating separate cognitive domains rather than cognitive performance in general could be useful to shed light on the relationship between Lp(a) and cognition.

Worse performance in tests assessing executive functions and processing speed have already been found to be related to vascular disease in patients with mild cognitive impairment (MCI) when compared to patients without vascular disease^58^. Notably, executive functions and processing speed have also been shown to be associated with inflammatory markers in BASE-II^59^. Pro-inflammatory effects of Lp(a) have been reported before^49,60^, which a mechanism that may also influence cognition, regarding the association between Lp(a) and the domains of executive functions and processing speed found in the current study^48,59^.

Similar to our approach, by examining the relationships between serum lipids and cognitive functions, Ancelin *et al*. defined four cognitive domains, visual memory, verbal fluency, psychomotor speed and executive abilities^7^ and demonstrated a poorer cognitive performance in men and women with a hypercholesterolemic pattern in general. In contrast to our study they did not specifically test for the effect of Lp(a).

Further research of the links and mechanisms between Lp(a) and cognition is desirable, because a medical Lp(a) modulation could be used as a treatment to prevent or decelerate age-related cognitive decline. With new lipid lowering approaches treatments such as PCSK9 inhibitors (Protein convertase subtilisin/kexin type 9) or antisense oligonucleotides that block the production of Lp(a) in the liver, the reduction of Lp(a) might result in better cognitive function^61–63^. Two studies investigated the relationship between AD or cognitive decline and their putative risk associated with PCSK9 inhibitors. However, no effect on cognitive performance was detected in these studies^50,51,64,65^. Sub-analysis in such large-scale studies might be of interest to detect Lp(a)-cognition association.

## Conclusion

In conclusion, we found a weak evidence for an association between Lp(a) plasma concentrations and cognitive performance in the male group only. However, our results suggest, that the cognitive domain of executive functions and processing speed might be of interest for future research based on the observed association with Lp(a) found here, e.g. in larger cohorts with longitudinal data. With respect to the current literature, the results of our study are plausible and controversial at the same time. Future studies using a longitudinal design and new Lp(a)-lowering target drugs may shed light on the potential mechanisms. Assessment of specific cognitive domains in vascular dementia research could represent a promising approach to detect specific and cognitive changes at an early stage of disease development.

## Supplementary information

Supplementary Information.
