# Supplementary material for: Relationship between Lipoprotein (a) and cognitive function – Results from the Berlin Aging Study II
Source: Sci Rep. 2020 Jun 30;10:10636. doi: 10.1038/s41598-020-66783-3 (PMC7326928; doi:10.1038/s41598-020-66783-3)
Supplement: Supplementary file 1 — Supplementary Information. [file 41598_2020_66783_MOESM1_ESM.pdf]

Supplementary Tables

Relationship between Lipoprotein (a) and cognitive function – Results from the Berlin Aging Study II

Franziska Röhr, Nina Bucholtz, Sarah Töpfer, Kristina Norman, Dominik Spira, Elisabeth Steinhagen-Thiessen, Christina M. Lill, Lars Bertram, Ilja Demuth, Nikolaus Buchmann and Sandra Düzel

Supplementary Table 1: Characteristics of the BASE-II study population according to Lp(a) in men

Men (n=659)			
	Lp(a) quintiles 2-5	Lp(a) quintile1	p-value
Age [years]	68 ± 4	68 ± 4	n.s.
Weight [kg]	83.41 ± 11.81	85.86 ± 13.65	n.s.
Height [cm]	175.59 ± 6.41	175.41 ± 5.45	n.s.
BMI [kg/m2]	27.05 ± 3.51	27.88 ± 4.06	0.046
Regular alkohol intake	464 (90.4)	143 (93.1)	n.s.
Current smoking	57 (11.1)	16 (11.1)	n.s.
Physically inactive	48 (9.4)	25 (17.4)	n.s.
GDS- score	24.61 ± 1.05	24.61 ± 1.01	n.s.
**Cognitive function**			
Verbal memory	-0.2133 ± 0.9781	-0.0644 ± 0.7825	n.s.
Visuo-construction	0.1364 ± 0.9799	0.1802 ± 0.8831	n.s.
Executive functions and processing speed	0.0852 ± 0.9898	-0.0728 ± 0.7425	n.s.
Verbal fluency	-0.0881 ± 0.7612	-0.1490 ± 0.6363	n.s.
**Lipid profile**			
Total Cholesterol [mg/dl]	204 ± 38	197 ± 35	0.027
HDL- C [mg/dL]	56 ± 14	53 ± 14	0.023
LDL - C [mg/dL]	125 ± 35	118 ± 31	0.023
TG [mg/dL]	119 ± 75	132 ± 82	0.017
Lp(a) [mg/L]	290.9 ± 339.3	15.3 ± 2.2	< 0.001
***APOE* genotype**			
22	2 (0.4)	3 (2.1)	n.s.
23	61 (11.9)	28 (19.3)	n.s.
24	11 (2.1)	4 (2.8)	n.s.
33	317 (61.7)	77 (53.1)	n.s.
34	115 (22.4)	31 (21.4)	n.s.
44	8 (1.6)	2 (1.4)	n.s.
Vitamin B12 [ng/L]	370.3 ± 214.5	377.1 ± 210.8	n.s.

Homocysteine [μmol/L]	14.11 ± 3.72	14.92 ± 4.79	n.s.
C-reactive protein [mg/L]	1.7 ± 2.4	2 ± 2.5	n.s.
Folic acid [μg/L]	11.04 ± 5.64	10.28 ± 4.08	n.s.
HbA1c [%]	5.7 ± 0.6	5.7 ± 0.6	n.s.
TSH basal [mU/L]	1.99 ± 1.72	2.48 ± 5.67	n.s.
Sodium [mmol/L]	139 ± 3	139 ± 3	n.s.
Potassium [mmol/L]	4.5 ± 0.4	4.5 ± 0.4	n.s.
Magnesium [mmol/L]	0.82 ± 0.07	0.82 ± 0.07	n.s.

BMI = body mass index; GDS = geriatric depression scale; HDL-C = high density lipoprotein cholesterol; LDL-C = low density lipoprotein cholesterol; Lp(a) = lipoprotein(a), *APOE*= apolipoprotein E; CRP = C-reactive protein; HbA1c =hemoglobin A 1c; TSH = thyroid stimulating hormone

Supplementary Table 2: Characteristics of the BASE-II study population according to Lp(a) in women

Woman (n=721)			
	Lp(a) quintiles 2-5	Lp(a) quintile 1	p- value
Age [years]	68 ± 3	67 ± 3	n.s.
Weight [kg]	69.71 ± 12.5	70.05 ± 11.61	n.s.
Height [cm]	162.95 ± 6.01	162.27 ± 6.04	n.s.
BMI [kg/m2]	26.27 ± 4.66	26.64 ± 4.45	n.s.
Regular alkohol intake	515 (87.3)	118 (90.1)	n.s.
Current smoking	57 (9.7)	6 (4.6)	n.s.
Physically inactive	44 (7.5)	12 (9.2)	n.s.
GDS score	24.60 ± 1.03	24.73 ± 1.11	n.s.
**Cognitive function (factor scores)**			
Verbal memory	0.2198 ± 0.8847	0.2534 ± 0.8334	n.s.
Visuo-construction	-0.1333 ± 0.9310	-0.1016 ± 0.9727	n.s.
Executive functions and processing speed	-0.0514 ± 0.8571	-0.1578 ± 0.6541	n.s.
Verbal fluency	0,1043 ± 0.6975	0.0905 ± 0.7485	n.s.
**Lipid profile**			
Total cholesterol [mg/dL]	227 ± 37	222 ± 41	n.s.
HDL- C [mg/dL]	69 ± 16	69 ± 17	n.s.
LDL- C [mg/dL]	138 ± 35	131 ± 38	0.049
TG[mg/dL]	103 ± 47	110 ± 65	n.s.
Lp(a) [mg/L]	320.4 ± 350.8	15.3 ± 2.1	< 0.001
***APOE* genotypes**			
22	3 (0.5)	0 (0)	n.s.
23	86 (14.6)	16 (12.2)	n.s.
24	19 (3.2)	6 (4.6)	n.s.
33	362 (61.4)	79 (60.3)	n.s.
34	110 (18.6)	28 (21.4)	n.s.
44	10 (1.7)	2 (1.5)	n.s.
Vitamin B12 [ng/L]	428.9 ± 314.7	397.3 ± 169.8	n.s.
Homocysteine [µmol/L]	12.51 ± 3.45	12.4 ± 3.45	n.s.
C-reactive protein [mg/L]	2.2 ± 3.4	2.1 ± 3.4	n.s.
Folic acid [µg/L]	11.9 ± 5.81	12.26 ± 5.6	n.s.
HbA1c [%]	5.6 ± 0.5	5.6 ± 0.6	n.s.
TSH basal [mU/L]	2.35 ± 4.59	2.03 ± 1.56	n.s.
Sodium [mmol/L]	140 ± 3	140 ± 3	n.s.
Potassium [mmol/L]	4.5 ± 0.4	4.5 ± 0.3	n.s.

Magnesium [mmol/L]	0.81 ± 0.06	0.81 ± 0.09	n.s.

BMI = body mass index; GDS = geriatric depression scale; HDL-C = high density lipoprotein cholesterol; LDL-C = low density lipoprotein cholesterol; Lp(a) = lipoprotein(a), APOE = apolipoprotein E; CRP = C-reactive protein; HbA1c =hemoglobin A 1c; TSH = thyroidstimulating hormone

Supplementary Table 3: Association between Lp(a) and cognitive domains

		Men				Women		
Factor score	beta	SE	p-value	R2*	beta	SE	p-value	R2*
Verbal memory	0.128	0.099	0.196	0.015	0.029	0.097	0.765	0.031
Visuo-construction	0.050	0.103	0.630	-0.013	-0.011	0.103	0.916	0.013
Executive functions and processing speed	-0.217	0.098	0.027	0.050	-0.110	0.089	0.215	0.039
Verbal fluency	-0.052	0.079	0.511	-0.010	0.044	0.078	0.571	0.007

The models were adjusted for: age, weight, height, HbA1c, *APOE* status, regular alcohol intake, current smoking, physical inactivity, GDS score, TSH, homocysteine, CRP, vitamin B12, magnesium, potassium, sodium, folic acid, morbidity index
*= R^2 corrected, p-values < 0.05 = statistical significant, corrected p-value (Bonferroni) < 0.0125 = statistical significant

Supplementary Table 4: Association between Lp(a) with *executive functions and processing speed* according to sex in subjects without *APOE4* genotype

		m				w		
	beta	SE	p	R^{2*}	beta	SE	p	R^{2*}
Model 1	-0.178	0.88	0.043	0.049	-0.093	0.080	0.246	0.035
Model 2	-0.202	0.092	0.029	0.056	-0.087	0.084	0.301	0.040
Model 3	-0.217	0.098	0.027	0.050	-0.010	0.089	0.215	0.039
Model 4	-0.221	0.098	0.024	0.050	-0.110	0.089	0.219	0.038
Model 5	-0.217	0.098	0.027	0.048	-0.124	0.088	0.159	0.054
Model 6	-0.213	0.098	0.029	0.049	-0.104	0.089	0.244	0.041

Model 1: weight, *APOE* genotype, age, HbA1c, height

Model 2: Model 1 + regular alcohol intake, current smoking, TSH, physical inactivity, homocysteine, CRP, GDS score

Model 3: Model 2 + vitamin B12, magnesium, potassium, sodium, folic acid, morbidity index

Model 4: Model 3 + HDL-C

Model 5: Model 3 + LDL-C

Model 6: Model 3 + TG

GDS = geriatric depression scale; HDL-C = high density lipoprotein cholesterol; LDL-C = low density lipoprotein cholesterol; Lp(a) = lipoprotein(a), *APOE* = apolipoprotein E; CRP = C-reactive protein; HbA1c =hemoglobin A 1c; TSH = thyroid stimulating hormone
*= R^2 corrected, p-values < 0.05 = statistical significant, corrected p-value (Bonferroni) < 0.0125 = statistical significant

Supplementary Table 5: Association of Lp(a) levels and cognitive function – an overview of the (inconsistent) study results from the literature

Cohort	Title	Study size	Age (years)	Cognitive assessment	Findings	Ref.
Italian longitudinal Study on Aging (ILSA)	Lipoprotein (a) and cognitive performances in an elderly white population	N=435	65-84	- MMSE<br>- Babcock Short Story<br>- Matrix Test	No association with cognition	[1]
Asian (Japanese) cohort	Dual inverse effects of lipoprotein(a) on the dementia process in Japanese late-onset Alzheimer´s disease	N=346	65-96	- MMSE<br>- NINCDS-ADRSA<br>- NINDS-AIREN<br>- brain computed tomography	- protective of AD<br>- increased risk of VD	[2]
Asian (Japanese) cohort	Lipoprotein(a) phenotypes in patients with vascular dementia	N=108	VD:66 ± 12.6<br>AD:67.1 ± 13.5<br>healthy: 65.5 ± 13.3	- DSM-IV<br>- NINCDS-ADRSA	- increased risk of VD	[3]
Kuopio Ischemic Heart Disease study (KIHD; Finland)	Is lipoprotein (a) protective of dementia?	N=2,532	42-61	- MMSE<br>- Geriatric Mental State	-protective effect on dementia in general in middle-aged men (only examined in men)	[4]
Caucasian (Italian) cohort	Lipoprotein(a),apolipoprotein E genotype, and risk of Alzheimer´s disease	N=124	With AD:<br>71.36 (9.55)<br>without AD:<br>67.67 (10.66)	-NINCDS-ADRSA	-age dependency:<br>increased risk of AD ⩽ 72 years, but a protective effect of AD with participants ⩾72 years	[5]

Abbreviations
VD: vascular dementia
AD: Alzheimer´s disease
MMSE = Mini Mental State Examination
Babcock Short Story = Babcock Short Story Recall Test
Matrix Test = Visual Search Matrix Test
NINCDS-ADRSA = National Institute of Neurological and Communicative Disorders and Stroke and the Alzheimer´s Disease and Related Disorders Association
NINDS-AIREN = National Institute of Neurologic Disorders and Stroke and Association Internationale pour la Recherche et l´Enseignement en Neurosiences
DSM-IV = Diagnostic and Statistical Manual of Mental Disorders
Geriatric Mental State

Supplementary Table 6. Model fit of results of PCA on eleven CERAD-Plus tests including 1, 2, 3, 4 and 5 factors

numbers of factors	χ^2	df	p	RMSEA	CFI
1	752.481	44	<.001	.126	.593
2	418.949	34	<.001	.106	.779
3	141.713	25	<.001	.068	.933
4	26.132	17	.072	.023	.995
5	6.809	10	.7433	<.001	>.999

Supplementary Table 7. Model fit of results of PCA on nine CERAD-Plus tests including 1, 2, 3, and 4 factors

numbers of factors	χ^2	df	p	RMSEA	CFI
1	689.002	27	<.001	.155	.587
2	387.682	19	<.001	.138	.770
3	107.793	12	<.001	.089	.940
4	9.176	6	.164	.023	.998

References

1. Sarti, C., et al., *Lipoprotein (a) and Cognitive Performances in an Elderly White Population: Cross-sectional and Follow-up Data.* Stroke: Journal of the American Heart Association, 2001. **32**(7): p. 1678-1683.
2. IWAMOTO, T., et al., *Dual inverse effects of lipoprotein (a) on the dementia process in Japanese late‐onset Alzheimer's disease.* Psychogeriatrics, 2004. **4**(3): p. 64-71.
3. Urakami, K., et al., *Lipoprotein (a) phenotypes in patients with vascular dementia.* Dementia and geriatric cognitive disorders, 2000. **11**(3): p. 135-138.
4. Kunutsor, S.K., et al., *Is lipoprotein (a) protective of dementia?* European journal of epidemiology, 2016. **31**(11): p. 1149-1152.
5. Solfrizzi, V., et al., *Lipoprotein (a), apolipoprotein E genotype, and risk of Alzheimer's disease.* Journal of Neurology, Neurosurgery & Psychiatry, 2002. **72**(6): p. 732-736.
